# Turpentine as an External Application for Small-Pox

**Published:** 1879-09

**Authors:** 


Turpentine as an External Application in Small-pox.—Dr Farr, of Lambeth, ascribes great value to turpentine as an external application in small-pox. He claims that it at once relieves any smarting or irritation, effectually corrects the unpleasant odor given off in the more confluent form of the disease, and seems, in a marked degree, to arrest pustulation, thereby modifying and sometimes entirely preventing pitting. In consequence of its powerful antiseptic and disinfectant properties, it tends, moreover, to prevent the spread of the infection. Mr. Farr uses it in the proportion of one part of rectified spirits of turpentine to three or four of olive oil, and applies it night and morning by means of a feather.—*Lancet.*

